# 4409 Indiana Clinical & Translational Science Monon Collaborative – Community Impact Hubs

**DOI:** 10.1017/cts.2020.273

**Published:** 2020-07-29

**Authors:** Sarah Wiehe, David Craig, Matthew Wilcox, Emily Hardwick, Carrie Lawrence, Fiona Schicho, Brenda Hudson

**Affiliations:** 1 Indiana University School of Medicine; 2 IUPUI; 3 Indiana Clinical and Translational Sciences Institute; 4 Indiana University

## Abstract

OBJECTIVES/GOALS:

- Conduct an environmental scan of Marion County (Indianapolis) neighborhoods using electronic medical record data, state health data, and social and economic data
- Develop strong network of community collaborators
- Conduct a thorough assessment for each targeted neighborhood by listening and understanding the pressing health issues in the community and working together to design and deliver solutions

METHODS/STUDY POPULATION:

- Identify measures in the 3 domains of vulnerability, health and assets for the targeted neighborhoods and conduct bivariate descriptive statistics and multivariable regression analyses to investigate association between measures of vulnerability and health outcomes.
- Initiate relationships with leaders and residents in targeted neighborhoods
- Locate organizations working in targeted neighborhoods through online mapping software and word-of-mouth at neighborhood events, and created a spreadsheet with contact information.
- Conduct multidisciplinary assessment (i.e. key informant interviews, focus groups, town hall meetings) of the targeted neighborhood.
- Iteratively synthesize assessments to develop areas of interest and relevance to the community.
- Develop a road map for solutions identified by the community.

RESULTS/ANTICIPATED RESULTS: The results from the environmental scan conducted will be displayed in a report and visual “map” of health outcomes and health determinants, including assets and barriers for the targeted neighborhoods. The research team will use results from the environmental scan coupled with listening activities including attendance at community events, key informant interviews and focus groups to develop relationships and strong collaborations with the targeted neighborhood stakeholders. The relationship building between the research team and community will provide increased trust and engagement that will further enhance the effectiveness of the assessments completed with the targeted neighborhood. The assessments will help to empower communities to develop sustainable solutions and drive future work that will lead to future grant applications and larger-scale implementation in other community impact hub neighborhoods. DISCUSSION/SIGNIFICANCE OF IMPACT: Through the community impact hub work, we will develop collaborative efforts with targeted neighborhoods with the greatest health inequities in the Marion County area. In partnership with these neighborhoods, we will build a foundation – a network of community collaborators and a focused plan – upon which we will improve the health outcomes of residents while learning best practices on how to do so.

